# Reply to Fujimoto, T. Comment on “Okaniwa, S. How Can We Manage Gallbladder Lesions by Transabdominal Ultrasound? *Diagnostics* 2021, *11*, 784”

**DOI:** 10.3390/diagnostics14020167

**Published:** 2024-01-11

**Authors:** Shinji Okaniwa

**Affiliations:** Department of Gastroenterology, Iida Municipal Hospital 1, Iida 395-8502, Nagano, Japan; okaniwa@cocoa.ocn.ne.jp; Tel.: +81-265-21-1255; Fax: +81-265-21-1266

Thank you for your interesting comments.

Since this article has targeted the differential diagnosis of gallbladder lesions and the differential diagnosis of T1 or shallow T2 GBC is still controversial, I only mentioned that sessile GPLs with an intact outer hyperechoic layer include not only non-neoplastic GPLs but also GBCs confined to the mucosa (T1a) or muscularis propria (T1b) or that have invaded the fibrous layer of the subserosa (T2).

I think your hypothesis is interesting. However, the conically thickened outermost hyperechogenic layer and deep hypoechoic areas have been reported in only a few case reports, and the frequency, sensitivity, and specificity in large numbers of cases do not seem to have been established. I think review articles should cite ultrasound findings with well-known frequency, sensitivity, and specificity in each disease, and hopefully these data will be clarified.

In another review that you pointed out [1], I only mentioned that a hypoechoic area within the deeper part of GBC with an intact outer hyperechoic layer may suggest invasion into the shallow fibrous layer of the lower serosa but did not cite it as a definite finding.

Contrast enhanced US (CEUS) has several advantages, including the diagnosis of biliary sludge, improved detection of microcystic structures in protruded lesions, and increased Doppler sensitivity. In addition, this review describes that CEUS is very useful in observing the continuity of the GB wall.

These studies are based on EUS; Hashimoto et al. [2] reported that CE-EUS provides enhanced visualization of the layered structure of the GB wall and could be valuable for predicting the depth of invasion. Imazu et al. [3] reported that CH-EUS could show the depth of invasion of biliary cancer (including GBC) more clearly than conventional EUS.

Therefore, I believe that CEUS could also provide more precise visualization of the layered structure of the GB wall, and CEUS with a high-frequency transducer could be valuable for predicting the depth of invasion.

Dr Fujimoto’s Comment has been revised based on Professor Okaniwa’s Reply [4].
